# Accuracy, readability, and bias of GPT-4o mini responses to oculoplastic patient questions

**DOI:** 10.3389/fopht.2026.1718017

**Published:** 2026-09-17

**Authors:** Terry Henry, Grace Staes, Alexius Russell, Nikisha Q. Richards-Walker

**Affiliations:** 1 Virginia Commonwealth University (VCU) Medical Center, Richmond, VA, United States; 2 Howard University College of Medicine, Washington, DC, United States; 3 Uniformed Services University of the Health Sciences, Bethesda, MD, United States

**Keywords:** Artificial intelligence, large language models, GPT-4o mini, oculoplastic surgery, ophthalmology, patient education, readability

## Abstract

**Purpose:**

This cross-sectional evaluation of model outputs evaluates the accuracy, readability, transparency, objectivity, completeness, and bias of GPT-4o mini responses to common oculoplastic patient questions.

**Methods:**

The study was reviewed and deemed exempt from institutional review board approval. Thirty-three questions were developed across eight clinical domains: floppy eyelid syndrome, cicatricial ectropion, entropion, blepharoplasty, facial aging, brow lift, ptosis, and facial nerve function. Seven domains contained four patient-centered questions, while the facial aging domain contained five questions, for a total of 33 questions. On September 16, 2024, GPT-4o mini was prompted for each question with a standardized prompt requesting responses from the perspective of an expert oculofacial surgeon, using the latest medical guidelines. Verbatim responses were recorded. Six American Society of Ophthalmic Plastic and Reconstructive Surgery fellowship-trained surgeons independently evaluated each response using a 5-point Likert scale assessing clinical accuracy, completeness, transparency, objectivity, and bias. Readability was assessed using established readability indices.

**Results:**

GPT-4o mini demonstrated moderate clinical accuracy and completeness. Clinical accuracy was rated favorably in 51% of ratings (101/198), while completeness was rated favorably in 42% (84/198). Transparency and objectivity demonstrated stronger performance. 78% of ratings (155/198) agreed or strongly agreed that responses were transparent and 76% of ratings (150/198) agreed or strongly agreed that responses were objective. 8% of ratings (16/198) agreed or strongly agreed that Bias was present, while 65% (129/198) disagreed or strongly disagreed. Readability analyses demonstrated that ratings required college- to graduate-level reading ability.

**Conclusion:**

GPT-4o mini generated transparent and objective ratings with low measured bias but demonstrated limitations in clinical accuracy, completeness, and readability. These findings apply specifically to GPT-4o mini and should not be generalized to other large language models. Future investigations should compare contemporary language models, evaluate alternative prompting strategies, and investigate multimodal systems that integrate text, clinical photographs, periocular anatomy, and patient symptoms.

## Introduction

Large language models (LLMs) have rapidly emerged as powerful tools for medical writing, education, and patient communication. Their ability to generate natural-language responses has led to growing interest in their use for patient education, clinical documentation, medical decision support, and communication between patients and healthcare professionals. Although these models can rapidly generate detailed responses to complex medical questions, their widespread availability has raised important concerns regarding clinical accuracy, readability, transparency, bias, and patient safety. Recent reviews suggest that LLMs have considerable potential to improve patient education; however, significant challenges remain regarding the reliability and accessibility of the information they generate (1, 2).

Health literacy is a critical determinant of ophthalmic outcomes. Limited health literacy has been associated with reduced treatment adherence, poorer clinical outcomes, and lower rates of preventive eye care and follow-up. Despite increasing efforts to improve patient education, many ophthalmic educational materials continue to exceed recommended reading levels, limiting their usefulness for a substantial proportion of patients (3, 4). Large language models have generated considerable interest as potential tools for producing more accessible patient education materials. However, improving readability alone is insufficient if responses sacrifice clinical accuracy, completeness, or patient safety. Consequently, expert evaluation of AI-generated educational content remains essential before these tools can be incorporated into routine clinical practice.

Oculoplastic surgery is an especially valuable specialty for evaluating AI-generated patient education, as many patient questions involve both functional and cosmetic concerns. Patients commonly seek information regarding eyelid position, tearing, ocular comfort, facial aging, periocular appearance, postoperative recovery, and surgical risk. While responses may appear comprehensive, they may fail to recognize important warning signs, omit critical treatment considerations, or oversimplify complex clinical decision-making. Furthermore, many oculoplastic disorders require individualized evaluation based upon physical examination, periocular anatomy, facial symmetry, and patient-specific goals, making expert review particularly important.

Previous investigations have examined the performance of large language models across numerous medical specialties. In dentistry, ChatGPT-generated responses demonstrated generally useful information but continued to exhibit important inaccuracies and omissions (5). Similar findings have been reported in urology, where investigators identified both promising educational applications and significant limitations for patient counseling (6). In plastic surgery, large language models have demonstrated value in academic writing and educational support, although concerns about hallucinations, incomplete sourcing, and inaccurate medical content remain significant (7).

Within ophthalmology, research evaluating large language models continues to expand. Recent studies have demonstrated encouraging performance in ophthalmic education, patient communication, and clinical question answering, while also highlighting persistent concerns about accuracy, reproducibility, readability, and hallucinations (8–10). In oculoplastics specifically, Al-Sharif and colleagues demonstrated that ChatGPT generally outperformed Google Bard when answering common oculoplastic patient questions, although both systems continued to require physician oversight (11). Similarly, Balas et al. found that ChatGPT-4 produced generally accurate responses regarding orbital and oculofacial disorders but generated text requiring relatively advanced reading ability (12). Cohen et al. further compared Google and ChatGPT responses to oculoplastics-related patient questions and their ability to generate customized patient education materials, demonstrating the potential utility of generative AI for patient education within the subspecialty (13).

Building upon this prior work, the present study specifically evaluates GPT-4o mini, a lightweight, cost-efficient language model, across eight common oculoplastic clinical domains. In addition to clinical accuracy and readability, the present investigation incorporates independent assessment of completeness, transparency, objectivity, and bias by a panel of six ASOPRS fellowship-trained surgeons. This focused evaluation therefore provides a multidimensional assessment of GPT-4o mini as a potential patient education resource within oculoplastic surgery.

Emerging multimodal large language models may further expand the role of artificial intelligence within ophthalmology. Many oculoplastic conditions, including ptosis, ectropion, entropion, facial nerve dysfunction, brow ptosis, eyelid asymmetry, and thyroid eye disease, depend heavily upon visual assessment of periocular anatomy and facial dynamics that cannot be adequately conveyed through text alone. Recent multimodal systems integrating clinical photographs with patient history have demonstrated improved diagnostic performance across multiple ophthalmic subspecialties, suggesting that future AI systems may better approximate real-world ophthalmic decision-making (14–18). Nevertheless, the present study intentionally evaluated a text-only application because text-based patient education remains the most common current use of publicly available large language models.

GPT-4o mini represents a lightweight, cost-efficient language model developed by OpenAI that supports both text and image inputs through the application programming interface; however, only text-based prompting was evaluated in this investigation (19). Accordingly, the present study should be interpreted as a focused evaluation of one lightweight language model rather than a comprehensive assessment of ChatGPT, GPT-4o, GPT-4.1, Claude, Gemini, DeepSeek, or other contemporary large language models.

The purpose of this study was to evaluate the clinical accuracy, completeness, transparency, objectivity, readability, and bias of GPT-4o mini responses to common oculoplastic patient questions using independent evaluation by fellowship-trained oculoplastic surgeons. By systematically examining responses across eight common clinical domains, this study seeks to better define the strengths and current limitations of GPT-4o mini as a potential patient education resource within oculoplastic surgery.

## Methods

### Study design

This cross-sectional evaluation of model outputs evaluates the accuracy, completeness, transparency, objectivity, bias, and readability of GPT-4o mini responses to common oculoplastic patient questions. The study was reviewed and deemed exempt from institutional review board approval. Ethical review and written informed consent were not required because the study did not involve patient participants or private patient data.

### Question development

Thirty-three questions were developed across eight clinical domains representative of common conditions encountered in oculofacial practice: floppy eyelid syndrome, cicatricial ectropion, entropion, blepharoplasty, facial aging, brow lift, ptosis, and facial nerve function. Seven domains contained four patient-centered questions, while the facial aging domain contained five questions, for a total of 33 questions.

### Model and prompt

GPT-4o mini was selected for its broad public availability, computational efficiency, and support for multimodal applications via the OpenAI application programming interface (19). Although the model can process both text and image inputs, only text-based prompting was evaluated in the present study. On September 16, 2024, each question was submitted individually using the identical prompt:

“Please respond as an expert oculofacial surgeon, using the latest medical guidelines relevant to the field of oculoplastic surgery.”

Responses were recorded verbatim in Microsoft Word before being transferred into Google Forms for reviewer evaluation. A single standardized expert-oriented prompt was used for every question to ensure consistency across all responses. Alternative prompting strategies, including patient-centered, sixth-grade readability, risk-focused, and shared decision-making prompts, were intentionally not evaluated. Consequently, the findings reflect the model’s performance with a single prompting strategy and should not be generalized to other prompt designs.

### Reviewer panel

A panel of six American Society of Ophthalmic Plastic and Reconstructive Surgery fellowship-trained surgeons reviewed the responses. Each reviewer assessed the GPT-4o mini responses using a 5-point Likert scale. The scale was 1 = strongly agree, 2 = agree, 3 = neutral, 4 = disagree, and 5 = strongly disagree. Responses were reviewed for clinical accuracy, completeness, bias, transparency, and objectivity. Clinical accuracy assessed the extent to which responses were consistent with accepted oculoplastic knowledge and current standards of care. Completeness assessed whether responses addressed the key clinical concepts necessary to adequately answer the question. Bias assessed the presence of unsupported assumptions, inappropriate generalizations, or potentially misleading content. Transparency evaluated the degree to which responses clearly communicated their reasoning, limitations, and areas of uncertainty. Objectivity assessed whether responses presented information in a balanced manner and avoided unjustified preference for a single management approach when multiple reasonable options existed.

### Readability analysis

Readability was assessed with established reading indices. These included the Gunning Fog Index, Simple Measure of Gobbledygook, Coleman-Liau Index, Flesch Reading Ease Score, Flesch-Kincaid Grade Level, and Automated Readability Index. These scores were calculated only from the GPT-4o mini responses.

### Statistical review

Descriptive statistics were used to summarize reviewer ratings. Mean and median scores were calculated for each review domain. Cumulative agreement scores were also calculated by combining strongly agree and agree responses. Cumulative disagreement scores were calculated by combining disagree and strongly disagree responses. Neutral scores were excluded from the cumulative tendency analysis.

## Results

### Overall response review

GPT-4o mini generated responses to all 33 oculoplastic patient questions. Responses were evaluated across five domains: clinical accuracy, completeness, transparency, objectivity, and bias. Mean reviewer scores across individual survey questions are presented in **Figure 1**. Readability was analyzed separately using established readability indices.

**Figure 1:**
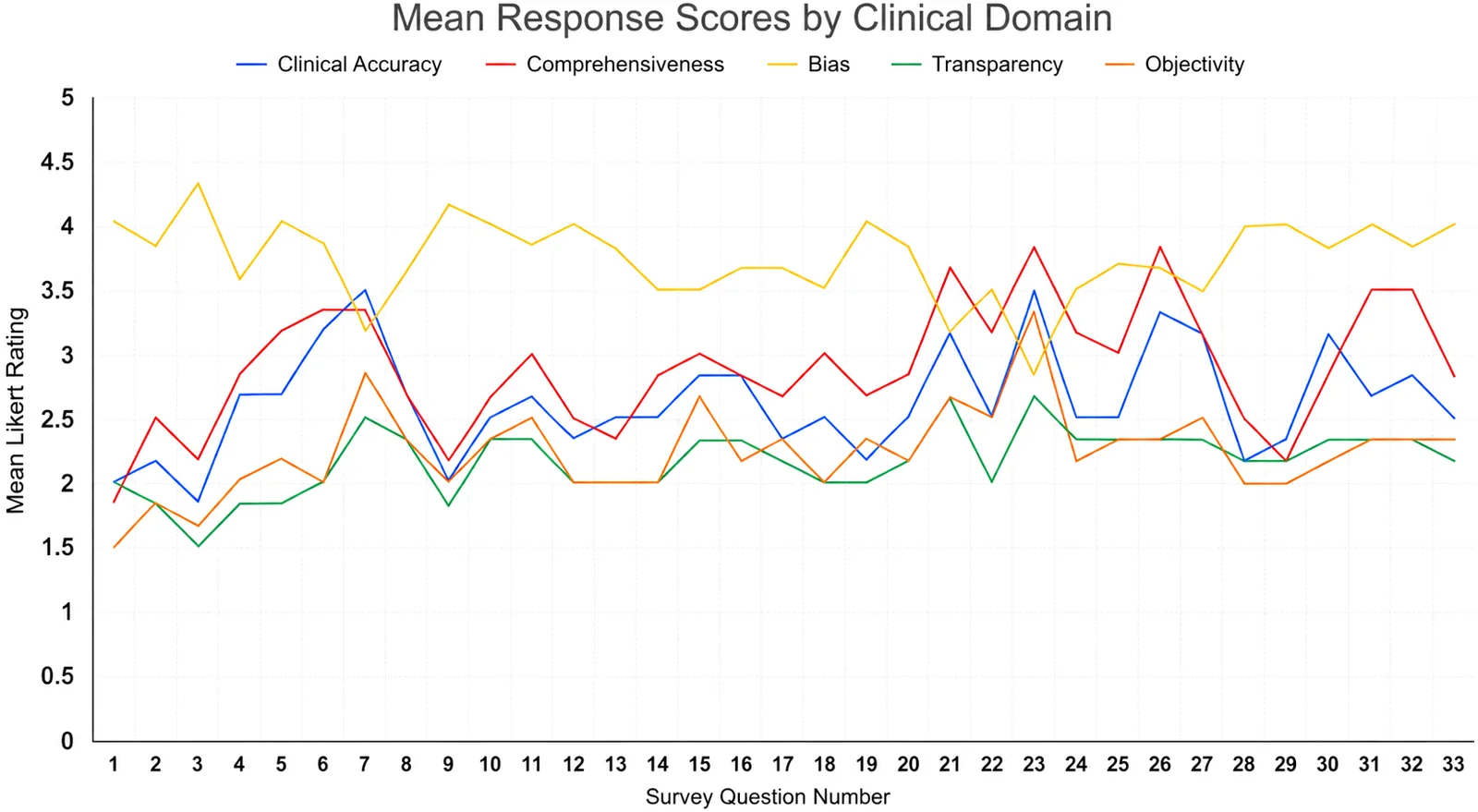
Mean reviewer scores for GPT-4o mini responses by survey question. Mean Likert scores for clinical accuracy, completeness, bias, transparency, and objectivity are presented across the evaluated oculoplastic patient questions. Lower scores indicate greater agreement with the assessed characteristic, based on the 5-point Likert scale (1 = strongly agree, 2 = agree, 3 = neutral, 4 = disagree, and 5 = strongly disagree).

Overall, responses were generally well organized, written in a professional tone, and frequently recommended consultation with an ophthalmologist or oculoplastic surgeon when appropriate. However, reviewers identified variable performance across clinical domains, with several responses lacking sufficient detail to support independent patient decision-making. The overall distribution of reviewer ratings across the five evaluation domains is presented in **Figure 2**.

**Figure 2:**
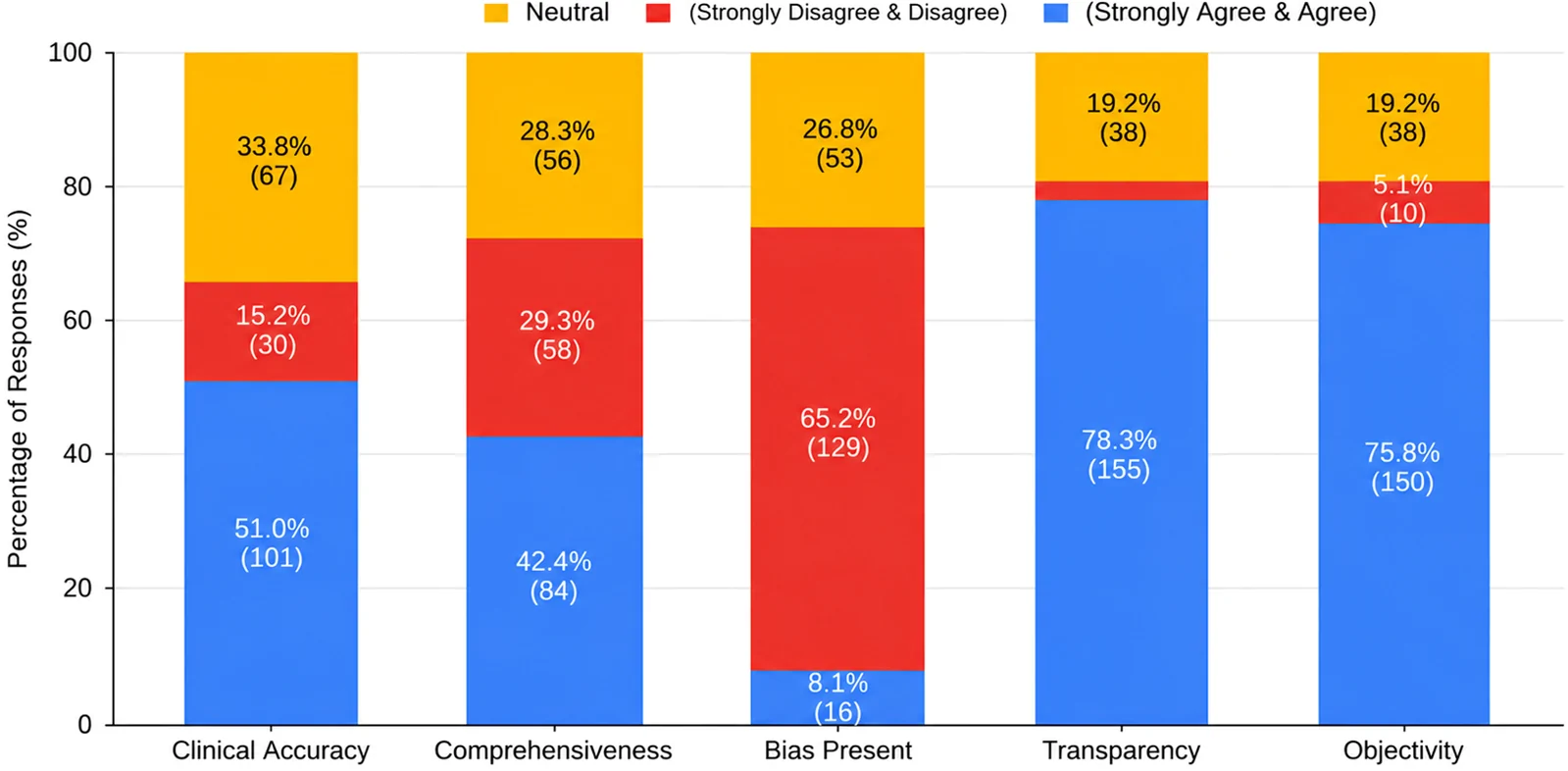
Distribution of reviewer ratings for GPT-4o mini responses across evaluation domains. Ratings from the six fellowship-trained oculoplastic surgeons are shown for clinical accuracy, completeness, bias, transparency, and objectivity. Ratings were recorded using a 5-point Likert scale ranging from strongly agree to strongly disagree.

### Clinical accuracy

Across all evaluated questions, the mean clinical accuracy score was 2.63. The median score was 2.5. These values show that responses were rated between “agree” and “neutral” for clinical accuracy. Independent reviewer analysis showed that 51% of ratings (101/198) agreed or strongly agreed that responses were clinically accurate. 15% of ratings (30/198) disagreed or strongly disagreed that responses were clinically accurate.

These findings suggest moderate accuracy. GPT-4o mini often gave reasonable general information. Still, some answers lacked detail about differential diagnosis, warning signs, or case-specific risks.

### Completeness

The mean completeness score was 2.89. The median score was 3. These scores show that responses were often rated near “neutral.” The independent reviewer’s analysis found that 42% of ratings (84/198) agreed or strongly agreed that responses were complete. 29% of ratings disagreed with the completeness.

This finding shows a major limit. The model could provide a useful overview, but many of the answers lacked sufficient depth. Oculoplastic patient questions often require details about risks, options, timing, and when urgent care is needed.

### Bias

The mean bias score was 3.73. The median score was 4. These values show a tendency toward disagreement with the presence of bias. 8% of ratings (16/198) agreed or strongly agreed that bias was present. Also, 65% of ratings (129/198) disagreed or strongly disagreed that bias was present. This suggests that overt bias was uncommon. However, low measured bias does not prove that bias was absent. Subtle bias can appear in word choice, treatment framing, appearance-based advice, or assumptions about patient goals.

### Transparency

The mean transparency score was 2.17. The median score was 2. Most responses were rated near “agree” for transparency. Independent reviewer analysis showed that 78% of ratings (155/198) agreed or strongly agreed that responses were transparent. This was one of the stronger areas. GPT-4o mini often gave clear explanations and described general limits. However, many responses did not cite sources or name the guidelines used. This limits the strength of the transparency finding.

### Objectivity

The mean objectivity score was 2.24. The median score was 2. These scores show that responses were often rated as objective. Independent reviewer analysis showed that 76% of ratings (150/198) agreed or strongly agreed that responses were objective. The model usually avoided strong unsupported claims. It also often describes several treatment options. Still, objectivity varied by topic. Cosmetic and reconstructive topics require extra care because patient goals may differ. Mean and median reviewer scores across the five evaluation domains are summarized in **Figure 3**.

**Figure 3:**
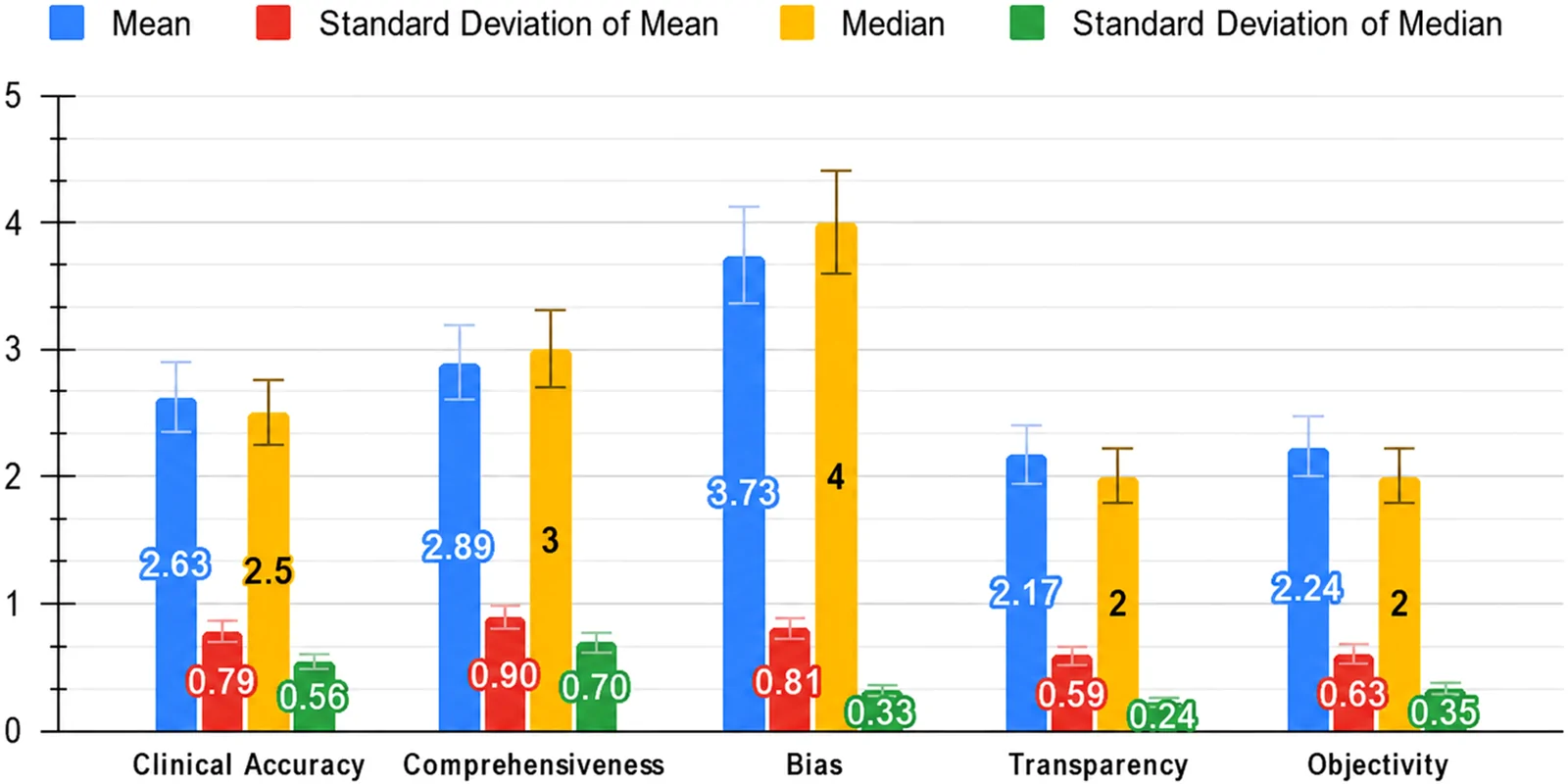
Comparison of mean and median reviewer Likert scores for GPT-4o mini responses across evaluation domains. Mean and median scores are shown for clinical accuracy, completeness, bias, transparency, and objectivity.

### Readability

Readability was a key concern. The Gunning Fog Index was 17.36, which suggests a graduate reading level. The SMOG score was 14.43, which suggests a college reading level. The Flesch Reading Ease Score was 19.6, which suggests very difficult text. The Flesch-Kincaid Grade Level was 14.1. The Automated Readability Index was 14.76. The Coleman-Liau Index was 17.15. These findings indicate that text generated by GPT-4o mini may present significant comprehension barriers for a substantial proportion of patients, underscoring the necessity for clear and accessible patient education materials. Prior work has also found that medical text from large language models can exceed common patient reading levels (20).

## Discussion

This study evaluated the performance of GPT-4o mini in answering common oculoplastic patient questions across eight clinical domains. Overall, GPT-4o mini demonstrated strengths in transparency, objectivity, and avoidance of overt bias while exhibiting moderate clinical accuracy and completeness. Readability emerged as the model’s greatest limitation, with responses consistently exceeding recommended reading levels for patient education materials. Collectively, these findings suggest that GPT-4o mini may serve as a useful adjunct for clinician-reviewed patient education but should not be considered a substitute for individualized evaluation by an ophthalmologist or oculoplastic surgeon.

### Clinical accuracy and completeness

Clinical accuracy and completeness remain fundamental requirements for any patient education resource. Although GPT-4o mini generated clinically appropriate responses for many questions, reviewers rated only 51% of ratings (101/198) were favorable for clinical accuracy, while 42% (84/198) were favorable for completeness. These findings suggest that the model frequently provided accurate introductory explanations but often omitted important details necessary for comprehensive patient counseling.

Several responses lacked discussion of differential diagnoses, indications for urgent evaluation, expected postoperative recovery, potential complications, or alternative management strategies. Such omissions are particularly relevant in oculoplastic surgery, where seemingly similar symptoms may represent markedly different disease processes requiring individualized management.

These findings are consistent with previous investigations evaluating large language models across multiple medical specialties. Studies in dentistry demonstrated that ChatGPT frequently generated helpful educational material while continuing to omit clinically important information (6). Similar observations have been reported in urology and surgical education, where AI-generated responses often provided reasonable overviews but required physician verification before clinical use (7, 8).

Within ophthalmology, recent investigations have similarly demonstrated encouraging performance while identifying important limitations. Al-Sharif et al. reported that ChatGPT generally outperformed Google Bard when answering oculoplastic patient questions, although physician oversight remained necessary (12). Likewise, Balas et al. found that ChatGPT-4 generated generally accurate responses regarding orbital and oculofacial disorders but continued to demonstrate important limitations in readability and completeness (13). The present study extends these findings by specifically evaluating GPT-4o mini using a standardized question set spanning eight common oculoplastic domains.

### Readability

Readability represented the most significant limitation identified in this study. Every readability index demonstrated that GPT-4o mini responses required college- or graduate-level reading ability, substantially exceeding the reading level recommended for patient education materials.

Health literacy is widely recognized as a critical determinant of health outcomes, with limited health literacy consistently associated with poorer understanding of disease, worse health status, and increased healthcare utilization (21, 22). In ophthalmology, where many chronic diseases require lifelong self-management, medication adherence, and regular follow-up, inadequate health literacy has been linked to poorer fund of knowledge, lower medication adherence, more missed appointments, greater visual field progression, and reduced participation in preventive eye care (3, 4). Therefore, patient-facing AI tools must provide information that is not only accurate but also accessible and understandable to individuals with varying levels of health literacy. Optimizing the readability of AI-generated educational materials may improve patient engagement, support informed decision-making, and ultimately contribute to better ophthalmic outcomes (23).

Recent investigations suggest that large language models may improve the readability of patient education materials when specifically prompted to simplify language. However, simplified outputs do not consistently achieve recommended reading levels and may occasionally sacrifice important clinical detail (4). The present study intentionally evaluated GPT-4o mini using an expert-oriented prompt rather than a readability-focused prompt. Consequently, the elevated reading levels observed may partially reflect the prompt design rather than inherent model limitations.

Future investigations should determine whether alternative prompting strategies can improve readability while preserving clinical accuracy and completeness.

### Transparency, objectivity, and patient trust

Transparency and objectivity represented the strongest-performing evaluation domains. GPT-4o mini generally communicated information logically, acknowledged the need for physician evaluation, and avoided presenting recommendations with unwarranted certainty. These characteristics likely contributed to the favorable reviewer ratings for transparency and objectivity.

Nevertheless, transparency extends beyond writing style. High-quality patient education should clearly communicate the evidence supporting recommendations, identify areas of uncertainty, and acknowledge limitations. GPT-4o mini rarely cited supporting evidence or identified the clinical guidelines that informed individual recommendations, limiting its ability to serve as an independent educational resource.

Trust represents another critical consideration for clinical AI adoption. Although enthusiasm for artificial intelligence continues to increase, both patients and physicians remain concerned regarding reliability, accountability, privacy, and safety. Recent ophthalmology literature demonstrates that clinician confidence in AI systems improves when model reasoning is transparent and clinically interpretable (24, 25). Accordingly, improving explainability may be equally important as improving accuracy when integrating large language models into ophthalmic practice.

### Bias and objectivity

Overt bias was uncommon within GPT-4o mini responses, with only 8% of ratings (16/198) agreed or strongly agreed that bias was present. Although encouraging, these findings should be interpreted cautiously. Oculoplastic surgery occupies a unique intersection between reconstructive and aesthetic medicine. Recommendations regarding eyelid position, facial aging, brow elevation, or cosmetic intervention are influenced not only by anatomy but also by individual patient preferences, cultural expectations, socioeconomic factors, and personal perceptions of appearance.

Recent reviews have emphasized that artificial intelligence systems trained using nonrepresentative datasets may inadvertently reinforce existing aesthetic norms, favor Western facial standards, or inadequately represent diverse ethnic and age-related facial characteristics (26–28). Even when overt bias is absent, subtle algorithmic preferences may influence language, framing, or treatment recommendations.

Emerging evidence suggests that these concerns extend beyond cosmetic preferences alone. Large language models have been shown to reproduce outdated race-based medical concepts and inaccurate clinical recommendations, reflecting biases embedded within their training data rather than current evidence-based practice (29). More broadly, algorithmic bias often stems from historical inequities and underrepresentation in clinical datasets, underscoring that equitable AI requires not only technical refinement but also representative data, multidisciplinary oversight, and ongoing auditing across diverse patient populations (30). Furthermore, recent evaluations have demonstrated that generative AI systems may produce confident but inaccurate medical information while inconsistently recognizing unsafe or inappropriate prompts, underscoring the importance of continued validation before these technologies are incorporated into routine patient counseling (31). Continued evaluation of fairness across diverse patient populations, particularly within oculoplastic surgery, remains essential before the widespread implementation of AI-generated cosmetic counseling.

### Hallucinations and patient safety

Although hallucinations were not specifically measured in this investigation, they remain an important consideration when evaluating large language models in medicine. Hallucinations refer to confident but factually incorrect or unsupported statements generated by language models. Recent ophthalmic benchmarking studies have demonstrated that hallucinations persist even among high-performing models and may contribute to misinformation or inappropriate clinical decision-making if responses are accepted without verification (32, 33).

Many of the deficiencies identified in the present study, including omission of important clinical details, incomplete counseling, and failure to discuss warning signs, highlight why physician oversight remains essential. Even when responses appear clinically plausible, patients should not rely upon AI-generated information without professional medical evaluation.

### Future role of multimodal artificial intelligence

The present investigation intentionally evaluated text-only responses. However, many oculoplastic disorders cannot be adequately assessed through text alone. Conditions such as ptosis, ectropion, entropion, facial nerve dysfunction, thyroid eye disease, eyelid asymmetry, and brow ptosis require direct observation of periocular anatomy, facial movement, and eyelid position. Multimodal AI systems that integrate photographs, imaging, and clinical history more closely resemble the diagnostic reasoning used by ophthalmologists in clinical practice. Recent studies have demonstrated that multimodal ophthalmic systems outperform text-only approaches across several ophthalmic applications (14–18). As multimodal models continue to mature, future investigations should evaluate whether integrating visual information improves diagnostic reasoning, educational quality, and patient counseling within oculoplastic surgery.

The adoption of multimodal AI should also be approached with caution. While text-based interactions require patients to disclose only the information they choose to provide, multimodal systems rely on the collection and processing of clinical photographs and other imaging data. As these technologies become more widely adopted, secure data governance, encryption, and privacy-preserving approaches such as federated learning will be important considerations, although federated learning itself does not completely eliminate the risk of information leakage (34, 35).

### Clinical implications

The findings of this study suggest that GPT-4o mini may have an important role as an adjunctive educational resource rather than an autonomous clinical decision-making system. Potential applications include assisting clinicians with drafting patient education materials, generating preliminary educational summaries before clinic visits, reinforcing postoperative instructions, and helping patients formulate questions before consultation. Artificial intelligence may also support shared decision-making by improving patient understanding and facilitating communication between physicians and patients.

Nevertheless, AI cannot replace the individualized clinical judgment required in oculoplastic surgery. Critical aspects of patient care, including physical examination, eyelid measurements, facial symmetry assessment, surgical planning, interpretation of imaging, and incorporation of patient values, remain dependent upon physician expertise (36). Rather than replacing clinicians, large language models should be viewed as tools that support communication while preserving the physician-patient relationship (37–39).

### Limitations

Several limitations should be acknowledged. First, only GPT-4o mini was evaluated; therefore, these findings should not be generalized to other contemporary large language models. Second, responses were generated at a single time point on September 16, 2024, approximately two years before the present manuscript revision. Because large language models undergo continuous updates, performance may change over time, and these findings may not reflect the current performance of GPT-4o mini.

Third, the study evaluated only 33 questions, limiting domain-specific conclusions. Fourth, only a single expert-oriented prompt was evaluated. Alternative prompting strategies emphasizing readability, shared decision-making, or risk communication may have produced different results. Fifth, although six fellowship-trained reviewers provided expert evaluation, subjective interpretation remains inherent to Likert-scale assessment. Finally, this investigation evaluated only text-based responses and therefore cannot assess the performance of multimodal AI systems that incorporate clinical images or other visual information.

### Future research

Future investigations should compare multiple contemporary large language models using standardized ophthalmic question sets to better characterize differences in clinical accuracy, readability, transparency, and reliability. Additional work should evaluate alternative prompting strategies designed to improve patient comprehension while preserving clinical accuracy.

Studies incorporating larger, multi-institutional panels of reviewers and patient evaluators would provide broader perspectives on educational quality and trustworthiness. Finally, multimodal investigations integrating clinical photographs, imaging, and patient-specific information may better define the future role of artificial intelligence within oculoplastic surgery.

## Conclusion

GPT-4o mini generated transparent and objective responses with low measured bias when answering common oculoplastic patient questions. However, its clinical accuracy and completeness were moderate, and its readability substantially exceeded recommended levels for patient education materials. These findings suggest that GPT-4o mini may serve as a useful adjunct for clinician-reviewed patient education but should not replace individualized clinical evaluation or physician judgment. Future work should compare contemporary language models, evaluate alternative prompting strategies, improve readability, and investigate multimodal AI systems that integrate visual and clinical information to better support patient-centered oculoplastic care.

## Data Availability

The original contributions presented in the study are included in the article/supplementary material. Further inquiries can be directed to the corresponding author.

## References

[1] Aydin S Karabacak M Vlachos V Margetis K . Large language models in patient education: a scoping review of applications in medicine. Front Med. (2024) 11:1477898. doi: 10.3389/fmed.2024.1477898 39534227 PMC11554522

[2] AlSammarraie A Househ M . The use of large language models in generating patient education materials: a scoping review. Acta Inform Med. (2025) 33(1):4–10. doi: 10.5455/aim.2024.33.4-10 40223858 PMC11986337

[3] Muir KW Lee PP . Health literacy and ophthalmic patient education. Surv Ophthalmol. (2010) 55(5):454–59. doi: 10.1016/j.survophthal.2010.03.005 20650503 PMC2918723

[4] Iskander M Hu G Coulon S Seixas AA McGowan R Al-Aswad LA . Health literacy and ophthalmology: a scoping review. Surv Ophthalmol. (2023) 68(1):78–103. doi: 10.1016/j.survophthal.2022.08.007 35995252

[5] Molena KF Macedo AP Ijaz A Carvalho FK Gallo MJD Wanderley Garcia de Paula E Silva F . Assessing the accuracy, completeness, and reliability of artificial intelligence-generated responses in dentistry: a pilot study evaluating the ChatGPT model. Cureus. (2024) 16(7):e65658. doi: 10.7759/cureus.65658 39205730 PMC11352766

[6] Solano C Tarazona N Angarita GP Medina AA Ruiz S Pedroza VM . ChatGPT in urology: bridging knowledge and practice for tomorrow's healthcare: a comprehensive review. J Endourol. (2024) 38(8):763–77. doi: 10.1089/end.2023.0700 38874270

[7] Wang AB Kim E Oleru O Seyidova N Taub PJ . Artificial intelligence in plastic surgery: ChatGPT as a tool to address disparities in health literacy. Plast Reconstr Surg. (2024) 153(6):1232e–1234e. doi: 10.1097/PRS.0000000000011202 37983817 PMC11090984

[8] Rengers TA Thiels CA Salehinejad H . Academic surgery in the era of large language models: a review. JAMA Surg. (2024) 159(4):445–50. doi: 10.1001/jamasurg.2023.6496 38353991

[9] Alqudah AA Aleshawi AJ Baker M Alnajjar Z Ayasrah I Ta'ani Y . Evaluating accuracy and reproducibility of ChatGPT responses to patient-based questions in ophthalmology: an observational study. Medicine (Baltimore). (2024) 103(32):e39120. doi: 10.1097/MD.0000000000039120 39121263 PMC11315477

[10] Chen X Zhao Z Zhang W Xu P Wu Y Xu M . EyeGPT for patient inquiries and medical education: development and validation of an ophthalmology large language model. J Med Internet Res. (2024) 26:e60063. doi: 10.2196/60063 39661433 PMC11669878

[11] Al-Sharif EM Penteado RC Dib El Jalbout N Topilow NJ Shoji MK Kikkawa DO . Evaluating the accuracy of ChatGPT and Google Bard in fielding oculoplastic patient queries: a comparative study on artificial versus human intelligence. Ophthalmic Plast Reconstr Surg. (2024) 40(3):303–11. doi: 10.1097/IOP.0000000000002567 38215452

[12] Balas M Janic A Daigle P Nijhawan N Hussain A Gill H . Evaluating ChatGPT on orbital and oculofacial disorders: accuracy and readability insights. Ophthalmic Plast Reconstr Surg. (2024) 40(2):217–22. doi: 10.1097/IOP.0000000000002552 37989540

[13] Cohen SA Yadlapalli N Tijerina JD Alabiad CR Chang JR Kinde B . Comparing the ability of Google and ChatGPT to accurately respond to oculoplastics-related patient questions and generate customized oculoplastics patient education materials. Clin Ophthalmol. (2024) 18:2647–55. doi: 10.2147/OPTH.S480222 39323727 PMC11423829

[14] Artiaga JCM Guevarra MCB Sosuan GMN Agnihotri AP Nagel ID Kalaw FGP . Large language models in ophthalmology: a scoping review on their utility for clinicians, researchers, patients, and educators. Eye. (2025) 39(15):2752–61. doi: 10.1038/s41433-025-03935-7 40855372 PMC12494959

[15] Kim BR Choi JY Yoo TK . Multimodal large language models as assistance for evaluation of thyroid-associated ophthalmopathy. Comput Biol Med. (2025) 192:110301. doi: 10.1016/j.compbiomed.2025.110301 40315721

[16] Ma R Cheng Q Yao J Peng Z Yan M Lu J . Multimodal machine learning enables AI chatbot to diagnose ophthalmic diseases and provide high-quality medical responses. NPJ Digit Med. (2025) 8:64. doi: 10.1038/s41746-025-01461-0 39870855 PMC11772878

[17] Jin K Yu T Grzybowski A . Multimodal artificial intelligence in ophthalmology: applications, challenges, and future directions. Surv Ophthalmol. (2026) 71:158–67. doi: 10.1016/j.survophthal.2025.07.003 40683606

[18] Vamsidhar D Kolhar S Patil S Kumar S . Advancements in ophthalmology healthcare using multimodal AI: a systematic review of methods, applications, and future directions. Discov Artif Intell. (2026) 6:236. doi: 10.1007/s44163-026-00980-330311153

[19] OpenAI . GPT-4o mini: Advancing cost-efficient intelligence (2024). Available online at: https://openai.com/index/gpt-4o-mini-advancing-cost-efficient-intelligence/ (Accessed May 30, 2026).

[20] Hershenhouse JS Mokhtar D Eppler MB Rodler S Storino Ramacciotti L Ganjavi C . Accuracy, readability, and understandability of large language models for prostate cancer information to the public. Prostate Cancer Prostatic Dis. (2025) 28:394–399. doi: 10.1038/s41391-024-00826-y 38744934 PMC12106072

[21] Ad Hoc Committee on Health Literacy for the Council on Scientific Affairs, American Medical Association . Health literacy: report of the Council on Scientific Affairs. JAMA. (1999) 281(6):552–7. doi: 10.1001/jama.281.6.552 10022112

[22] Juzych MS Randhawa S Shukairy A Kaushal P Gupta A Shalauta N . Functional health literacy in patients with glaucoma in urban settings. Arch Ophthalmol. (2008) 126(5):718–24. doi: 10.1001/archopht.126.5.718 18474786

[23] Cheng BT Tanna AP . Association of health literacy and health care utilization among glaucoma patients. J Glaucoma. (2023) 32(2):139–44. doi: 10.1097/IJG.0000000000002133 36223324

[24] Ghaffar F Islam YZ Furtado N Burns C . Trust and accuracy in AI: optometrists favor multimodal AI systems over unimodal for glaucoma diagnosis in collaborative environments. Comput Biol Med. (2025) 198:111132. doi: 10.1016/j.compbiomed.2025.111132 41027339

[25] Ran AR Lui CH Tham YC Cheng CY Lam CY Cheung WL . The acceptance of ophthalmic artificial intelligence for eye diseases: a literature review and qualitative analysis. Eye. (2025) 39:2353–62. doi: 10.1038/s41433-025-03878-z 40514441 PMC12325719

[26] Al-Dhubaibi MS Mohammed GF Atef LM Bahaj SS Al-Dhubaibi AM Bukhari AM . Artificial intelligence in aesthetic medicine: applications, challenges, and future directions. J Cosmet Dermatol. (2025) 24(6):e70241. doi: 10.1111/jocd.70241 40501296 PMC12159716

[27] Elder A Ring C Heitmiller K Gabriel Z Saedi N . The role of artificial intelligence in cosmetic dermatology—current, upcoming, and future trends. J Cosmet Dermatol. (2021) 20(1):48–52. doi: 10.1111/jocd.13797 33151612

[28] Frank K Day D Few J Chiranjiv C Gold M Sattler S . AI assistance in aesthetic medicine—a consensus on objective medical standards. J Cosmet Dermatol. (2024) 23(12):4110–5. doi: 10.1111/jocd.16481 39091136 PMC11626373

[29] Omiye JA Lester JC Spichak S Rotemberg V Daneshjou R . Large language models propagate race-based medicine. NPJ Digit Med. (2023) 6(1):195. doi: 10.1038/s41746-023-00939-z 37864012 PMC10589311

[30] Ferryman K Mackintosh M Ghassemi M . Considering biased data as informative artifacts in AI-assisted health care. N Engl J Med. (2023) 389(9):833–838. doi: 10.1056/NEJMra2214964 37646680

[31] Tiller NB Marcon AR Zenone M Kidd KE Jeukendrup AE Master Z . Generative artificial intelligence-driven chatbots and medical misinformation: an accuracy, referencing and readability audit. BMJ Open. (2026) 16(4):e112695. doi: 10.1136/bmjopen-2025-112695 41980854 PMC13158598

[32] Pan X Bai Y Zou K Zhou Y Zhou J Fu H . EH-Benchmark: ophthalmic hallucination benchmark and agent-driven top-down traceable reasoning workflow. Inf Fusion. (2026) 126:103631. doi: 10.1016/j.inffus.2025.10363142574925

[33] Sonmez SC Sevgi M Antaki F Huemer J Keane PA . Generative artificial intelligence in ophthalmology: current innovations, future applications and challenges. Br J Ophthalmol. (2024) 108(10):1335–1340. doi: 10.1136/bjo-2024-325458 38925907 PMC11503064

[34] Radeva MN Hristova E Georgiev RT Zlatarova ZI . Awareness, trust, and expectations of AI for glaucoma care among Bulgarian ophthalmologists: role of demographic factors. PLoS Digit Health. (2026) 5(1):e0001199. doi: 10.1371/journal.pdig.0001199 41569993 PMC12826508

[35] Sirin U Idreos S . Data storage and management for image AI pipelines. In: Companion of the 2025 International Conference on Management of Data (SIGMOD Companion '25). (2025). (New York, NY: Association for Computing Machinery). doi: 10.1145/3722212.3725640

[36] van Ooijen PMA Darzi E Dekker A . Data storage, cloud usage and artificial intelligence pipeline. In: De Cecco CN van Assen M Leiner T , editors. Artificial Intelligence in Cardiothoracic Imaging. (2022). (Cham: Springer). p. 45–57. doi: 10.1007/978-3-030-92087-6_5

[37] Tanya SM Nguyen AX Buchanan S Jackman CS . Development of a cloud-based clinical decision support system for ophthalmology triage using decision tree artificial intelligence. Ophthalmol Sci. (2023) 3(1):100231. doi: 10.1016/j.xops.2022.100231 36439697 PMC9692027

[38] Lorenzini G Arbelaez Ossa L Shaw DM Elger BS . Artificial intelligence and the doctor-patient relationship: expanding the paradigm of shared decision making. Bioethics. (2023) 37(5):424–429. doi: 10.1111/bioe.13158 36964989

[39] Abbasgholizadeh Rahimi S Cwintal M Huang Y Ghadiri P Grad R Poenaru D . Application of artificial intelligence in shared decision making: scoping review. JMIR Med Inform. (2022) 10(8):e36199. doi: 10.2196/36199 35943793 PMC9399841

